# Abdominal T_1_ relaxation times at 7T

**DOI:** 10.1007/s10334-025-01317-4

**Published:** 2026-01-03

**Authors:** Petr Bulanov, Petr Menshchikov, Johannes A. Grimm, Niklas Himburg, Simon Schmidt, Stephan Orzada, Mark E. Ladd, Sebastian Schmitter

**Affiliations:** 1 Division of Medical Physics in Radiology, German Cancer Research Center (DKFZ), Heidelberg, Germany; 2 Faculty of Physics and Astronomy, Heidelberg University, Heidelberg, Germany; 3 Medical Physics and Metrological Information Technology, Physikalisch-Technische Bundesanstalt (PTB), Braunschweig, Berlin, Germany; 4 Institut für Physik und Astronomie, Technische Universität Berlin, Straße des 17, Juni 135, 10623 Berlin, Germany; 5 Faculty of Medicine, Heidelberg University, Heidelberg, Germany; 6 Center for Magnetic Resonance Research, University of Minnesota, Minneapolis, MN USA

**Keywords:** 7T, T_1_ mapping, Abdomen T_1_ values, pTx, *B*_1_^+^ shimming, UHF

## Abstract

**Objective:**

To quantify the T_1_ relaxation times at 7T for key abdominal organs and tissues, including the liver, kidney, spleen, and skeletal paraspinal muscles.

**Materials and methods:**

T_1_ mapping was performed on a 7T whole-body scanner with a parallel transmission (pTx) system in five healthy volunteers. Static pTx (B_1_^+^ shimming) was applied to maximize B_1_^+^ magnitude in the target region. For each T_1_ map, data were collected using eight snapshot gradient-echo inversion recovery sequences during breath holding. Voxel-wise T_1_ values were calculated based on DICOM data using exponential model fitting.

**Results:**

Mean abdomen T_1_ values and corresponding coefficients of variation across the group were: 1829 ± 60 ms, 3.3% for the kidney cortex; 2619 ± 83 ms, 3.2% for the kidney medulla; 1378 ± 48 ms, 3.5% for the liver; 1954 ± 28 ms, 1.4% for the skeletal paraspinal muscle; 1770 ± 36 ms, 2.0% for the spleen.

**Conclusion:**

In this study, we quantified the T_1_ relaxation times at 7T for key abdominal organs and tissues, including the liver, kidney, spleen, and skeletal paraspinal muscle. To achieve this, pTx was applied to mitigate B_1_^+^ dropouts in the target regions. The B_1_^+^ insensitive snapshot gradient-echo inversion recovery method was utilized to acquire accurate and reproducible T_1_ maps.

**Supplementary Information:**

The online version contains supplementary material available at 10.1007/s10334-025-01317-4.

## Introduction

The T_1_ relaxation time is one of the key parameters in MRI that impacts signal and contrast in several ways. While T_1_ contrast generated by qualitative T_1_-weighted imaging (e.g., with and without contrast administration) is used in clinical practice to highlight pathologies [1, 2], quantitative T_1_ mapping [3] enables the direct measurement of tissue-specific T_1_ relaxation times. Tissue T_1_ values are important not only as a promising quantitative biomarker in cardiac [4–8], brain [9–14], abdominal [15–22], and other applications [23–26]. In addition, T_1_ relaxation values are an essential input for many simulation studies, modelling and corrections in quantitative imaging. For example, accurate T_1_ quantification is required in chemical exchange saturation transfer (CEST) to calculate relaxation-compensated metrics such as the apparent exchange-dependent relaxation (AREX) [27]. Similarly, T_1_ values are needed to correct certain rapid B_1_^+^ mapping methods such as the double-angle technique with short TR, where T_1_ dependence otherwise introduces a bias in the resulting B_1_^+^ map [28]. Furthermore, quantitative magnetization transfer (qMT) modeling also requires T_1_ values as input parameters [29]. Therefore, the knowledge of reference T_1_ values of healthy tissues is essential for those and many other applications.

T_1_ relaxation values, however, are strongly dependent on the magnetic field strength (B_0_) [30], with the tendency to increase with field strength. Although extensive literature exists providing quantitative assessments of T_1_ times across different human organs for 1.5 Tesla and 3 Tesla, reference data at 7Tesla remain incomplete. While mapping T_1_ at 7T has been successfully performed in the brain [31, 32], T_1_ mapping of the body faces several technical challenges: motion artifacts, strong B_0_/B_1_^+^ inhomogeneities (including total B_1_^+^ dropouts), and SAR restrictions. Moreover, most of the existing T_1_ mapping techniques exhibit a strong dependency on the B_1_^+^, requiring accurate B_1_^+^ correction [22]. Severe B_1_^+^ inhomogeneities can be crucial for such studies. Due to these reasons, only a limited number of T_1_ quantification studies at UHF have focused on the human body to date: (i) Li et al.[33] performed renal T_1_/T_2_ mapping using a magnetization-prepared single breath-hold fast spin echo sequence; (ii) Gajdošík et al. [34] applied MR spectroscopy to study liver T_1_ relaxometry using STEAM inversion recovery (IR); and (iii) Damen et al. [35] measured pancreatic T_1_ values using a Look-Locker IR turbo field echo sequence. To the best of the authors’ knowledge, there is presently no literature data providing T_1_ values of the human spleen at 7T or higher. Moreover, for other abdominal organs (liver, kidney, pancreas), only single studies exist. This highlights the need for further abdominal T_1_ relaxometry studies at 7T to establish reliable comparative values using robust and B_1_⁺ unbiased methods.

In this study, we quantified the T_1_ relaxation times at 7T for key abdominal organs and tissues, including the liver, kidney, spleen, and skeletal paraspinal muscles. T_1_ maps were acquired using a simple, robust and straightforward approach: an optimized gradient-echo inversion recovery (GRE-IR) technique that shows low sensitivity to variations of the B_1_⁺ field, which is an essential requirement for the quantification. Reduced acquisition time was achieved by using a snapshot-FLASH [36] readout: one IR pulse was applied for the acquisition of several k-space lines (i.e. segments), resulting in an in-vivo feasible protocol. The sensitivity to B_1_⁺ inhomogeneities and the accuracy of the measured T_1_ values were initially validated in a phantom study. To further mitigate B_1_⁺ dropouts in the target regions—ensuring sufficient SNR and effective inversion efficiency (IE)—static parallel transmission (pTx) technique, also termed B_1_^+^ shimming [37], was used [38].

## Materials and methods

### MRI data acquisition and analysis

MR examinations were performed on a 7T whole-body MR scanner (Magnetom 7T, Siemens Healthineers, Erlangen, Germany) equipped with an 8 × 1 kW pTx system and using a local 8-channel transmit/receive body coil [39].

#### B_1_^+^ mapping and static pTx

Channel-wise relative B_1_^+^ maps were obtained [40] during breath-holding at end-exhale using a low-flip angle GRE scan with the following parameters: resolution = 1.5 × 1.5 × 6 mm^3^; TE/TR = 3.1/6.1 ms; nominal FA = 15°; readout bandwidth (BW) = 501 Hz/px; acquisition time (TA) = 15 s. Subsequently, B_1_^+^ phase shimming (static pTx) was applied to locally maximize the B_1_^+^ efficiency, which maximizes the transmit field magnitude ($\left|{B}_{1}^{+}\right|$) per input power within a given ROI (approximately 5—15 cm in diameter), corresponding to the target organ. [41, 42]. This process was performed using a stand-alone computer and custom-built MATLAB software* B*_*1*_^+^ *phase shimming was performed by maximizing the transmit efficiency:*

$$\eta =\frac{\left(\left|{\sum }_{ch=1}^{8}{\widehat{B}}_{1,ch}^{+}{b}_{ch}\right|\right)}{\left({\sum }_{ch=1}^{8}\left|{\widehat{B}}_{1,ch}^{+}\right|\right)},$$

where ${\widehat{B}}_{1,ch}^{+}$ are the estimated *B*_*1*_^+^ maps of each channel ch and${b}_{ch}= {e}^{i{\phi }_{ch}}$ are the complex* RF* phases with channel*-*dependent transmit phases ${\phi }_{ch}$.

The final B_1_^+^ distribution after applying the B_1_^+^ shim was mapped using an MR fingerprinting (MRF)-based B_1_^+^ mapping technique [43] (parameters: resolution = 2 × 2 × 6 mm^3^; TE/TR = 2.35/4.7 ms; nominal FA = 40° (${FA}_{nom}$); BW = 590 Hz/px; TA = 40 s). Relative B_1_^+^ values (rB_1_) were defined as the ratio of the obtained *actual* ${FA}_{act}$ to the nominal ${FA}_{nom}$:

1 $$r{B}_{1}=\frac{{B}_{1,act}}{{B}_{1,nom}}=\frac{{FA}_{act}}{{FA}_{nom}}$$

#### B_0_ shimming and mapping

After acquiring localizer images and B_1_^+^ shimming, second-order B_0_ shimming was performed. If required, an additional manual frequency adjustment was applied by correcting the mean frequency offset within the ROI. The frequency offset was estimated from a modified B_0_ mapping acquisition originally proposed for UHF cardiovascular applications [44] (acquired during breath-holding at end-exhale, parameters: resolution = 1.5 × 1.5 × 6 mm^3^, TR = 100 ms; TE_1_ = 2.08, TE_2_ = 3.08; nominal FA = 12°; BW = 781 Hz/px; TA = 34 s).

#### T_1_ mapping

**Fig. 1:**
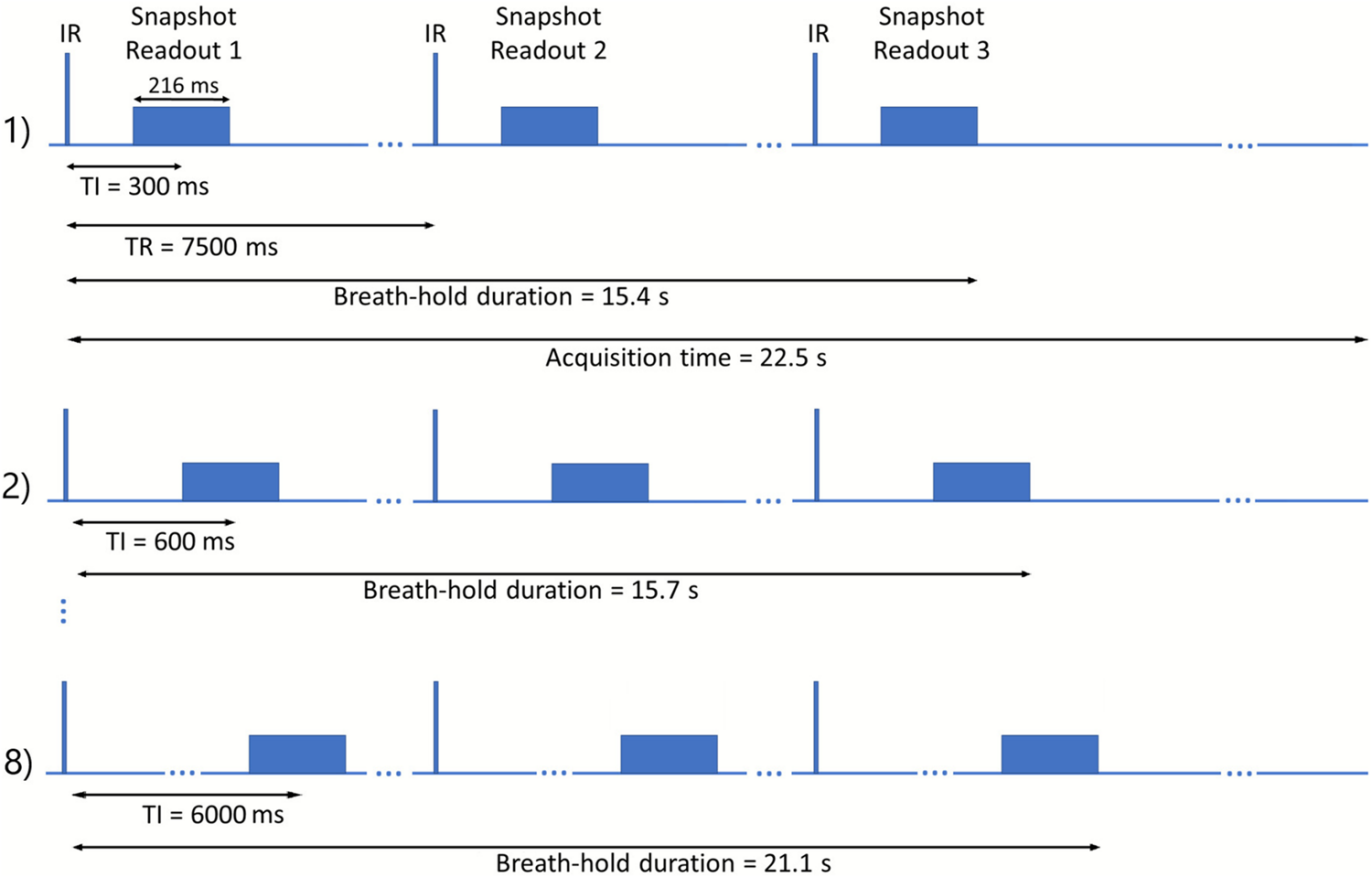
The pulse sequence consists of three shots with linearly reordered readouts following an IR and a TR of 7500 ms. Each snapshot includes 36 acquisitions with an echo spacing of 6 ms, resulting in a one-shot readout time of 216 ms. The inversion times (TI) are 300, 600, 1000, 2000, 3000, 4000, 5000, and 6000 ms, with TI remaining constant for each sequence. All acquisitions are performed during breath-hold. Acquiring a complete T_1_ map requires repeating the sequence eight times, each with a different TI. The total acquisition time for one sequence was 3xTR = 22.5 s, however the actual breath-hold duration depends on TI and ranges from 15.4 to 21.1 s (2 × TR + readout block duration/2 + TI = 15 s + 0.108 s + TI)

For each T1 map, data were collected using eight snapshot 2D GRE-IR sequences (c.f. Fig. 1) during breath-holding at end-exhale, which has been demonstrated to be a more stable respiratory position than inhalation [45], with the following parameters: standard non-adiabatic, non-selective sinc-shaped 180° IR pulse of 10,240 µs duration and amplitude of 175 V; 3 snapshot-FLASH readout-trains per slice, each acquiring 36 linear reordered k-space lines per shot, GRAPPA acceleration factor R = 2; TR = 7500 ms; nominal readout FA = 12°; readout echo-spacing = 6 ms; TE = 3 ms; number of slices = 1; resolution = 2 × 2 × 3 mm^3^; TI values = 300, 600, 1000, 2000, 3000, 4000, 5000, and 6000 ms. The TA for one sequence was 3xTR = 22.5 s, however the actual breath-hold duration depends on TI and ranges from 15.4 to 21.1 s (Fig. 1).

TA = 16–21 s, depending on TI. The total acquisition time for a complete set of eight images for one T1 map was approximately 6 min, including breath-hold instructions. The stability of the slice position was visually verified (by analysing liver size or/and intervertebral disc location) between scans with different TIs, and corrupted or non-fitting positioned acquisitions were repeated to ensure consistent positioning.

T_1_ map reconstruction was performed in MATLAB R2019b (MathWorks, Natick, MA) using DICOM images acquired with varying TIs. Voxel-wise T_1_ values were calculated using the following model fitting equation [3]:

2 $${S}_{n}=\left|a+b{e}^{-\frac{{TI}_{n}}{T1}}\right|,$$

where ${S}_{n}$ denotes the signal magnitude at different time points ${TI}_{n}$ and a, b denotes real-valued fitting parameters. All data with IR curve fitting R^2^ < 0.99 were filtered out. IE η was calculated as:

$${S}_{n}=\left|a(1-2\eta {e}^{-\frac{{TI}_{n}}{T1}})\right|.$$

η=-b/2a

Additionally, the M_z_ evolution, as well as IR curves in a Shepp–Logan phantom were simulated for T_1_ = 2000 ms and FA = 5°, 15°, and 30° (Supplementary Information Figs. S1 and S2).

### Study protocol for phantom

**Fig. 2:**
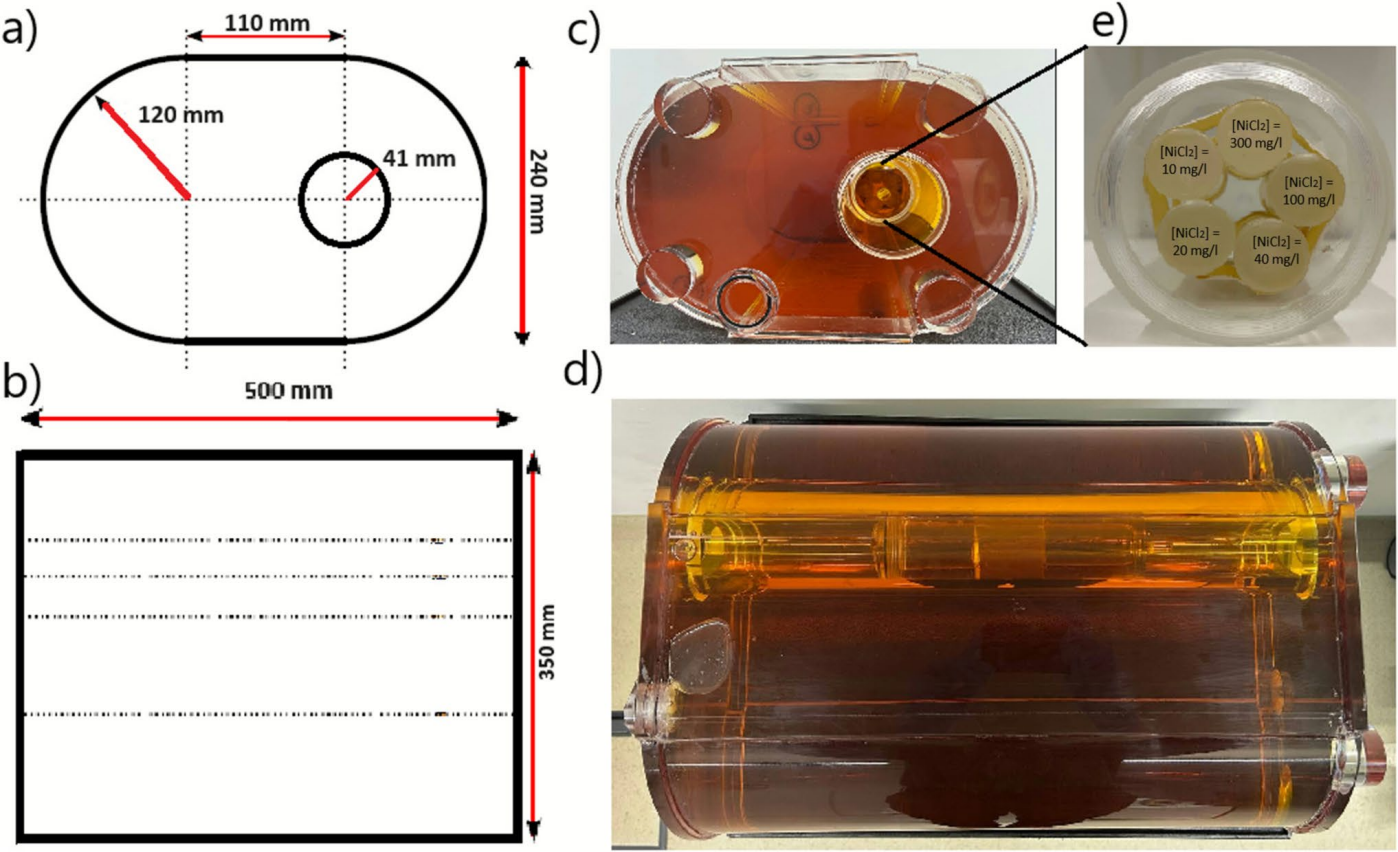
**a**–**d** Body-sized (length = 500 mm, height = 240 mm, width = 350 mm) torso phantom with an off-center tubular cut-out (82 mm inner diameter) in the head-foot direction and a smaller, cylindrical phantom (diameter = 72 mm, length = 200 mm) inserted into the tubular cut-out. e) Five tubes (25 ml each) were placed inside the inner cylindrical phantom containing NiCl_2_ water solutions with the following concentrations: 10, 20, 40, 100, 300 mg/l

In a first step, phantom measurements were performed using a body-sized PMMA torso phantom (length = 500 mm, height = 240 mm, width = 350 mm, volume = 35 L) shown in Fig. 2 to evaluate the robustness of the applied technique, especially with respect to B_1_^+^ variations. The phantom contains an off-center tubular cut-out (inner diameter = 82 mm) aligned along the head–foot direction, into which a smaller cylindrical phantom (diameter = 72 mm, length = 200 mm) was inserted (Fig. 2c–d). Both the main body and the inner cylinder were filled with a water-based solution containing 49% polyvinylpyrrolidone (PVP) and 1.8% NaCl, adjusted to mimic in vivo electrical properties with a relative permittivity of 44 and conductivity of 0.56 S/m at 300 MHz [46]. Five tubes (25 ml each) were placed inside the inner cylindrical phantom (Fig. 2e) and contained NiCl_2_ water solutions with the following concentrations: 10, 20, 40, 100, 300 mg/l.

To minimize temperature-related variations in T_1_ values, each phantom measurement was conducted at a fixed temperature of 20 °C, following 72 h of temperature stabilization in the MRI room with controlled air temperature. The study protocol for the phantom included B_0_ and B_1_^+^ shimming within the ROI corresponding to the inner cylindrical phantom with NiCl_2_ tubes. B_1_^+^ phase shimming was performed and subsequently the scanner’s reference voltage was adjusted to achieve a mean rB_1_ ≈ 1 within the corresponding ROI. GRE-IR T_1_ mapping was performed using the same protocol as for the subjects. Measurements were repeated five times to assess the standard deviations of the measured mean T_1_ values for each tube. Reference T_1_ values for each NiCl_2_ concentration were obtained using PRESS spectroscopy with the following parameters: TR = 8000 ms, TE = 20 ms, 4 averages, and 26 TIs from 50 to 6300 ms with a 250 ms increment. Spectra processing and approximation were performed using jMRUI 7.0 software. T_1_ values quantification from spectra data was carried out using Eq. 2.

Additionally, to investigate the dependence of the calculated T_1_ values from B_1_^+^ values, phantom T_1_ mapping was performed with three different reference transmitting voltages, providing mean (across all tubes) rB_1_ ≈ 0.6, rB_1_ ≈ 1, and rB_1_ ≈ 1.5.

### Study protocol for subjects

Five healthy volunteers (26 ± 3 years old; female/male ratio: 3/2) with normal weight BMI (20.6 ± 1.4) and without known fatty liver disease were recruited after approval by the ethics committee of the Medical Faculty Heidelberg, Germany (S-154/2014). All participants received a detailed explanation of the study and provided signed informed consent in accordance with institutional guidelines. All subject measurements were performed in the morning after a 10-h fasting period.

The study protocol for the subjects (total duration: approximately 18 min per organ) included B_0_ and B_1_^+^ shimming within the ROIs, which targeted specific anatomical regions. Eight GRE-IR sequences with varying TIs were applied with the appropriate B_0_ and B_1_^+^ shimming parameters. Data acquisition consisted of five datasets from the liver, kidney, spleen, and skeletal paraspinal muscles. T_1_ maps were retrospectively reconstructed from the DICOM data of the GRE-IR acquisitions. To evaluate the inter-subject variability of the T_1_ values, the coefficients of variation (COVs) were calculated as the ratio of the standard deviation to the absolute mean.

## Results

**Fig. 3:**
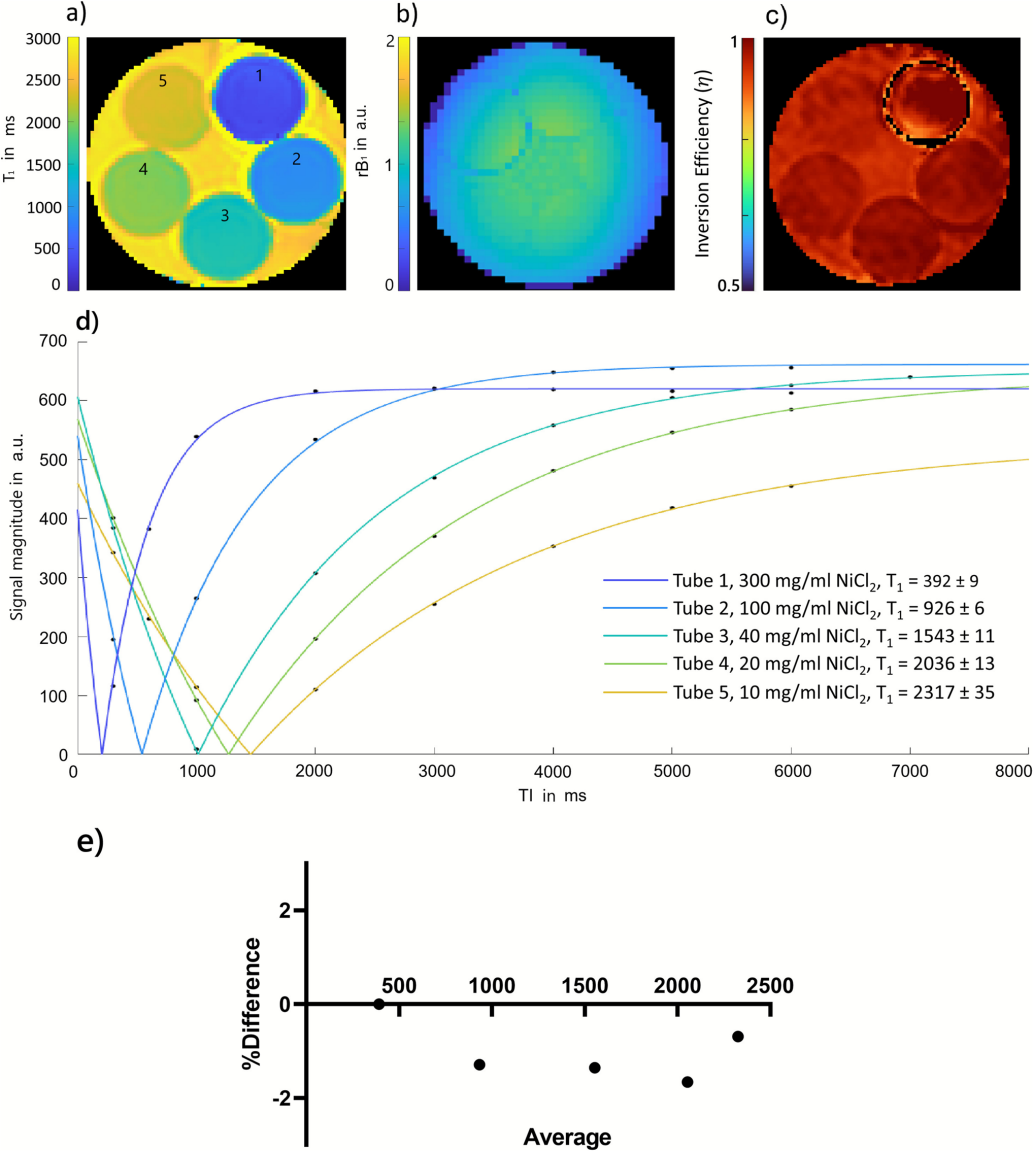
**a** Phantom T_1_ map and **b** relative B1 map obtained at 7T, **c** inversion efficiency map and **d** Inversion recovery curves for tubes with NiCl_2_ water solutions of different concentrations: 300, 100, 40, 20, 10 mg/l. Individual experimental data points are shown as circle markers, with approximation curves represented as colored lines. **e** Bland–Altman plot of percentage difference between measured T_1_ values and reference (measure with PRESS spectroscopy)

The T_1_ map obtained in the phantom (Fig. 3a) with an rB_1_ = 1.08 (mean value across all tubes) (Fig. 3b), illustrates low variability in T_1_ values within each individual tube, but high contrast in T_1_ values between tubes with different NiCl_2_ concentrations. High IE (Fig. 3c) of 0.96 ± 0.03 ensures accuracy of T_1_ mapping. Representative IR curves for each tube (Fig. 3d) show a high accuracy of the fitting process for each T_1_ value, with mean fitting R^2^ > 0.999. The obtained GRE-IR mean T_1_ values with SD across five measurements conducted at a fixed phantom temperature of 20 °C are summarized in Table 1 and Bland–Altman plot (Fig. 3e) in comparison to the reference values for all NiCl_2_ concentrations. The average percentage difference between the reference values and the measured values is 1.6%, demonstrating a high accuracy of the GRE-IR T_1_ mapping technique at 7T using static pTx. The GRE-IR T_1_ values across five measurements demonstrate strong reproducibility for all tubes with corresponding average COV of 1.2%.

**Table 1:** T_1_ relaxation times obtained at 7T with GRE-IR for the tubes with corresponding NiCl_2_ concentrations

Tube	[NiCl_2_], mg/l	Reference T_1_, ms	Measured T_1_, ms (mean ± SD)
1	300	392	392 ± 9
2	100	938	926 ± 6
3	40	1564	1543 ± 11
4	20	2070	2036 ± 13
5	10	2333	2317 ± 35

Phantom temperature during all measurements was fixed at 20 °C. PRESS spectroscopy results were used as reference T_1_ values

**Fig. 4:**
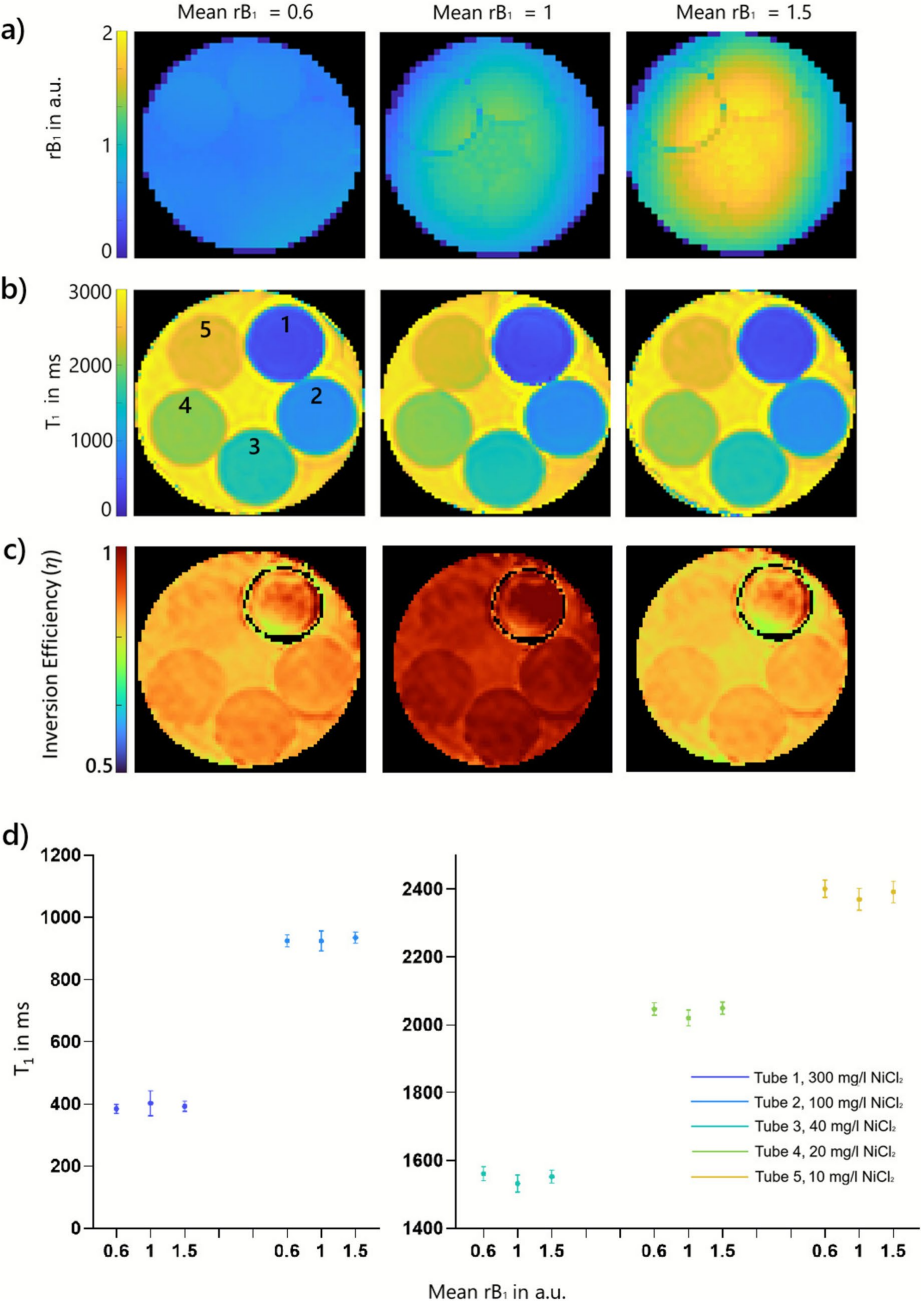
Three phantom datasets obtained with different reference transmitting voltages, corresponding to mean (across all tubes) rB_1_ = 0.6 (first column, “Mean rB_1_ = 0.6’’), mean rB_1_ = 1 (second column, “Mean rB_1_ = 1’’), and rB_1_ = 1.5 (third column, “Mean rB_1_ = 1.5’’). **a** Corresponding relative B_1_ maps demonstrate clearly visible differences in contrast, but **b** T_1_ maps have low variation in contrast across different rB_1_ independently from **c** IE. **d** Mean values with SD for each tube with different NiCl_2_ concentration (shown by different colors) for measurements with different mean rB_1_

Three phantom datasets, obtained with mean rB_1_ values measured across all tubes of 0.61 (Fig. 4, first column), 1.08 (Fig. 4, second column) and 1.52 (Fig. 4, third column) demonstrate low variation in the determined T_1_ values across these measurements (Fig. 4b). IE values (Fig. 4c) varied between measurements with different rB_1_ and were 0.83 ± 0.02, 0.96 ± 0.03 and 0.85 ± 0.02 correspondingly. The average percentage difference between mean T_1_ values (Fig. 4d) of 2% indicates that the fitting model (Eq. 2) is effective in mitigating the influence of IE and actual readout FA on T_1_ value quantification, underscoring the reliability of the proposed T_1_ mapping methodology.

**Fig. 5:**
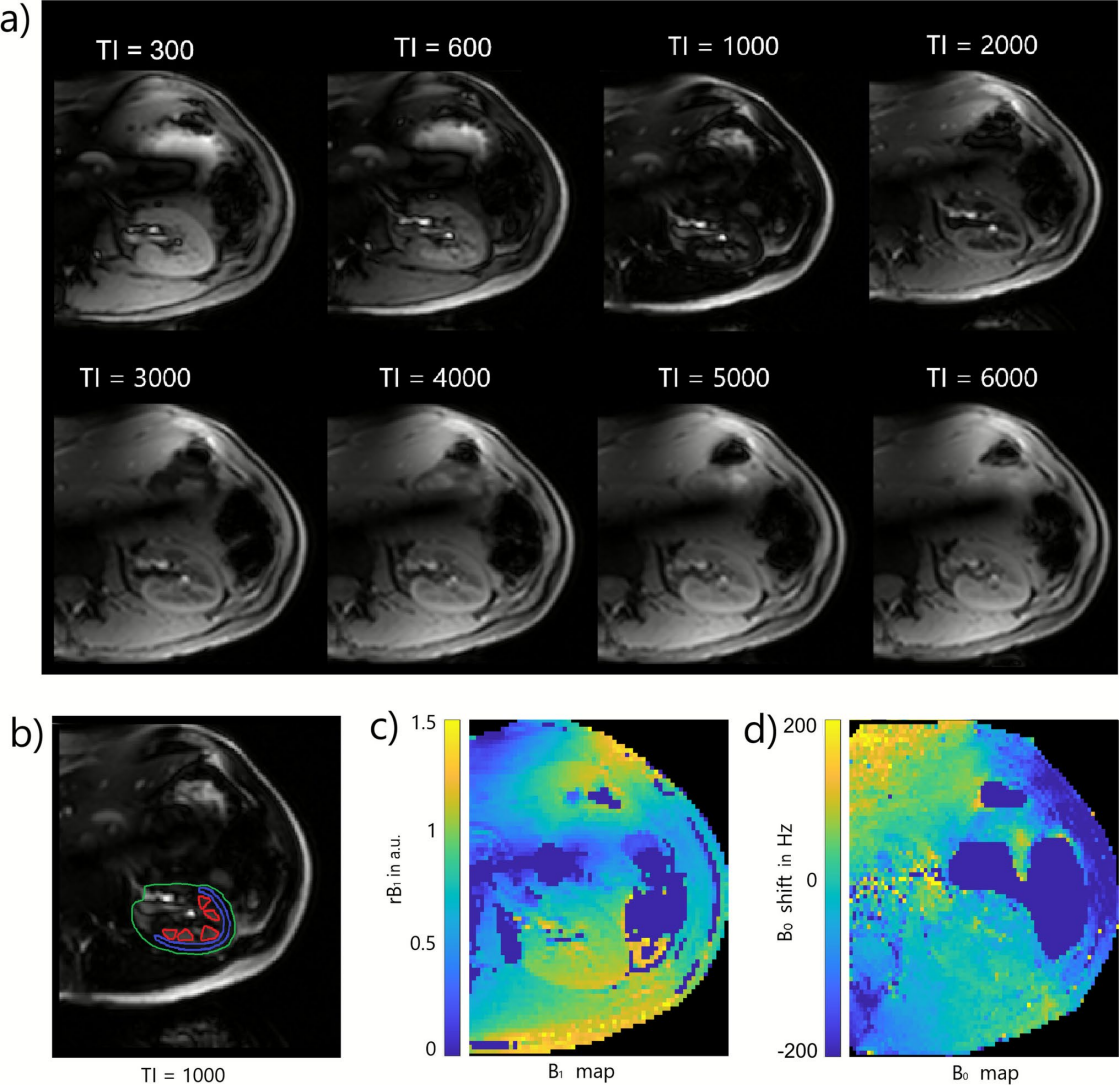
**a** GRE-IR images of the kidney obtained with different TIs (300–6000 ms). **b** ROI of medulla (red line) and cortex (blue line) are shown on image with TI = 1000 ms and high contrast between regions. The ROI used for B_1_^+^ shimming is shown by the green line. **c** Relative B_1_ map. **d** B_0_ map in Hz

GRE-IR images of the kidney acquired at various TIs (Fig. 5a) demonstrate high image quality in the kidney region, devoid of signal dropouts and artifacts within the ROI (outlined in green; Fig. 5b). The corresponding relative B_1_^+^ map (Fig. 5c) and B_0_ map (Fig. 5d) exhibit low variability across the kidney, with rB_1_ = 0.96 ± 0.13 and ∆B_0_ = -0.4 ± 25 Hz.

**Fig. 6:**
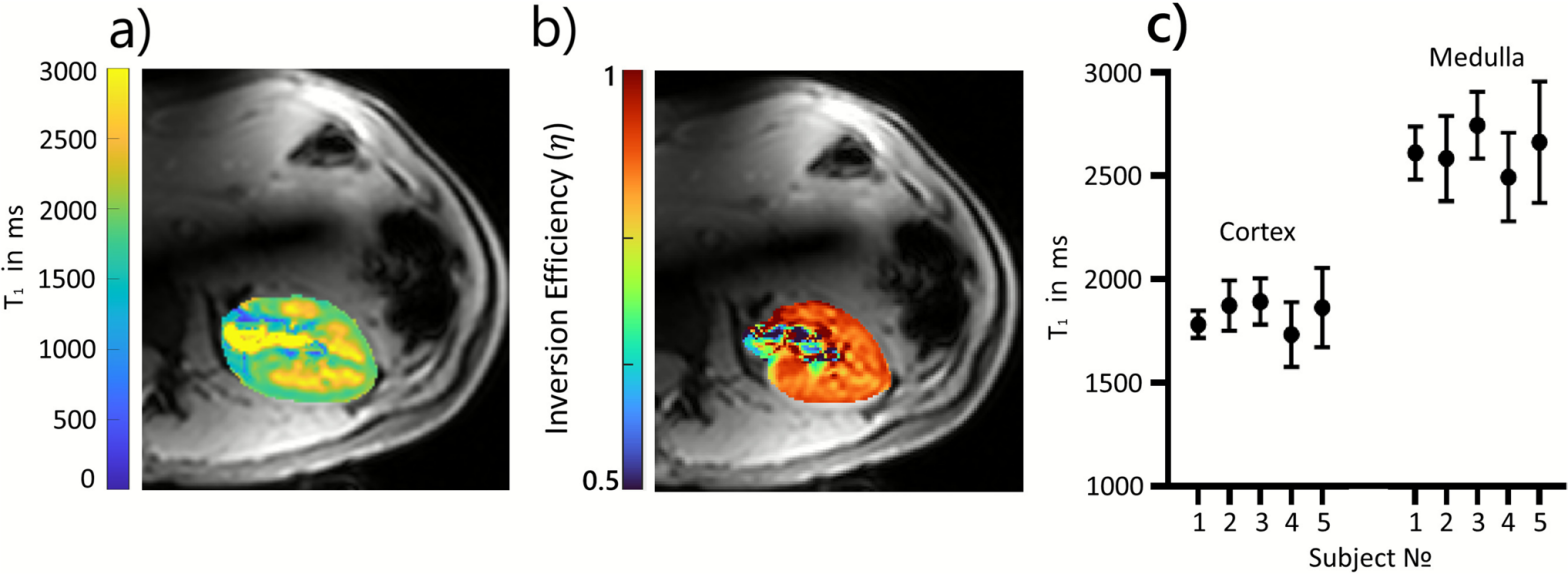
**a** Representative T_1_ map of the kidney, showing high contrast between the cortex and medulla. **b** Inversion efficiency across both regions, with an average of 0.89 ± 0.04. **c** Mean T_1_ values with standard deviations within the cortex and medulla across five subjects. T_1_ values exhibit low variability across all subjects, with coefficients of variation of 3.3% for the cortex and 3.2% for the medulla. The mean T_1_ values across the group are 1829 ± 60 ms for the cortex and 2619 ± 83 ms for the medulla

The kidney T_1_ map demonstrates the expected contrast between the kidney cortex and medulla (Fig. 6a). IE (Fig. 6b) exhibit low variability across both regions, with an average of 0.89 ± 0.04. Mean T_1_ values with standard deviations (SDs) for the cortex and medulla (outlined in blue and red, correspondingly; Fig. 5b) across five subjects (Fig. 6c) yield COVs of 3.3% for the cortex and 3.2% for the medulla. The mean T_1_ values across the group are 1829 ± 60 ms for the kidney cortex and 2619 ± 83 ms for the kidney medulla.

**Fig. 7:**
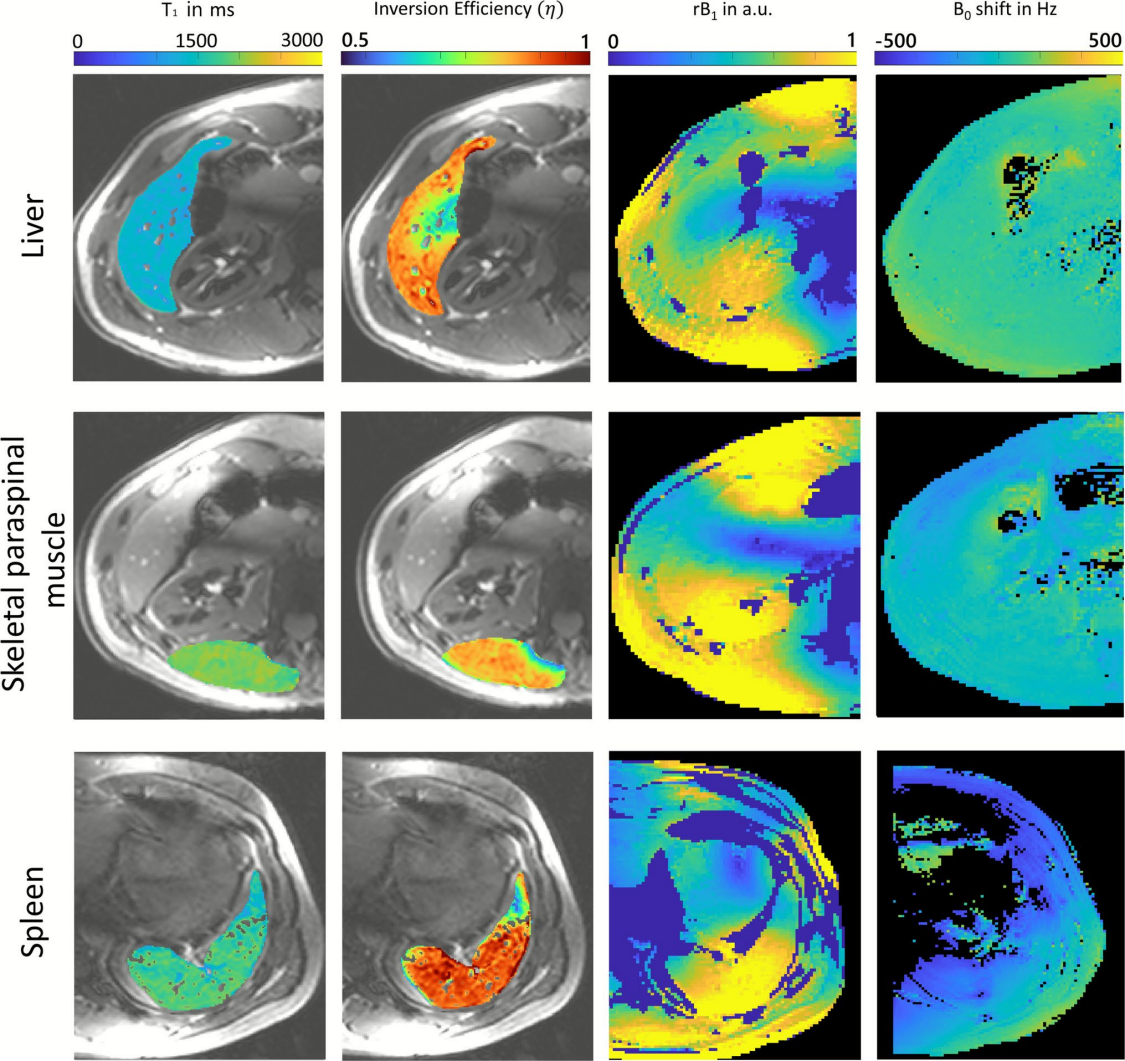
Liver (first row), skeletal paraspinal muscle (second row) and spleen (third row) representative T_1_ maps (first column), inversion efficiency maps (second row), relative B_1_^+^ maps (third column) and B_0_ maps (forth column)

**Fig. 8:**
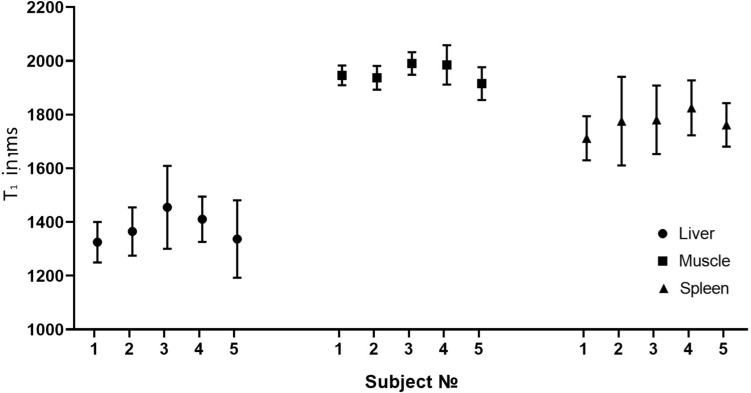
Mean T_1_ values with standard deviations across five subjects. T_1_ values exhibit low variability, with coefficients of variation of 3.5% for the liver, 2.5% for the skeletal paraspinal muscle, and 2.0% for the spleen. The mean T_1_ values across the group are: 1378 ± 48 ms for the liver, 1954 ± 28 ms for the skeletal paraspinal muscle, and 1770 ± 36 ms for the spleen

Representative T_1_ maps (Fig. 7, first column) of the liver, skeletal paraspinal muscle, and spleen show low variability of T_1_ values within each target volume. The IE maps (Fig. 7, second column) exhibit a strong visual correlation with the corresponding relative B1⁺ maps (Fig. 7, third column) and vary both across and within organs: 0.82 ± 0.09 for the liver, 0.83 ± 0.07 for skeletal paraspinal muscle, and 0.85 ± 0.09 for the spleen. Despite these variations, the T_1_ maps remain homogeneous and independent of IE, demonstrating the robustness of the approach to IE fluctuations. The corresponding relative B_1_⁺ values were 0.60 ± 0.13 for the liver, 0.85 ± 0.23 for skeletal paraspinal muscle, and 0.84 ± 0.21 for the spleen. The B_0_ maps (Fig. 7, last row) show good homogeneity across all target organs, supporting the reliability of the obtained results. Mean T_1_ values with SDs across five subjects (Fig. 8) exhibit COVs of 3.5% for the liver, 1.4% for the skeletal paraspinal muscle, and 2.0% for the spleen. The mean T_1_ values across the group are 1378 ± 48 ms for the liver, 1954 ± 28 ms for the skeletal paraspinal muscle, and 1770 ± 36 ms for the spleen.

All abdominal T_1_ relaxation times obtained in this study are summarized in Table 2 and compared to values reported in the literature, if available.

**Table 2:** T_1_ values of abdominal organs at 7T: this study and literature comparison

Body area	This study T_1_, ms (mean ± SD)	Literature T_1_, ms (mean ± SD)	Imaging method*	Number of subjects	References
Liver	1378 ± 48	1362 ± 83	STEAM	11	Gajdošík et al. [34]
Skeletal muscle**	1954 ± 28	1864 ± 243	SR-GRE	3	Marschar et al. [47]
Spleen***	1770 ± 36	–	–	–	–
Kidney cortex	1829 ± 60	1661 ± 68	FSE-IR	2	Li et al. [33]
Kidney medulla	2619 ± 83	2094 ± 67	FSE-IR	2	Li et al. [33]

*STEAM (STEAM spectroscopy); SR-GRE (saturation recovery GRE sequence with five non-selective 90° preparation pulses); FSE-IR (magnetization-prepared single-breath-hold fast spin echo)

**Skeletal muscles T_1_ values obtained in the calf

***We demonstrate spleen T_1_ values at 7T for the first time, to the best of our knowledge

## Discussion

In the present study, we measured T_1_ values of the human kidney, liver, spleen, and skeletal paraspinal muscle at 7T using static pTx and a B_1_^+^ insensitive IR-based acquisition technique. Notably, to the best of our knowledge, this is the first study to report spleen T_1_ values at 7T as well as of T_1_ values of the paraspinal skeletal muscle. Furthermore, while previous liver T_1_ quantification studies at 7T have been performed using MR spectroscopy [34], our work presents the first imaging-based demonstration of 2D liver T_1_ maps in humans at UHF.

The estimated liver T_1_ values in the present study (1378 ± 48 ms) are in good agreement with the previously reported values (1362 ± 83 ms) [34]. The close correspondence with spectroscopy-based measurements from Gajdošík et al. [34]—considered the gold standard for T_1_ relaxometry—supports the reliability of the T_1_ values obtained in our study. For the kidney, cortical T_1_ values (1829 ± 60 ms) are comparable to those reported by Li et al. (1661 ± 68 ms) [33]. However, the medullary T_1_ values observed in our study (2619 ± 83 ms) are 25% higher than those previously reported using a different approach (2094 ± 67 ms) [33], which encourages further investigation. Furthermore, our work reports for the first time T_1_ values of the paraspinal skeletal muscle (1954 ± 28 ms), and the obtained values are consistent with values obtained in the calf skeletal muscle (1864 ± 243 ms) [47].

The use of different techniques may also contribute to the variability in reported T_1_ values across studies, underscoring the challenges of reproducibility and standardization of T_1_ mapping [48–50]. Such challenges are augmented at UHF by significant B_1_^+^ inhomogeneities. While conventional IR imaging and MR spectroscopy methods are relatively robust to B_1_^+^ variations, many advanced and rapid T_1_ mapping techniques are more susceptible to these effects. In variable flip angle T_1_ mapping, B_1_^+^ inhomogeneities, e.g. spatial variation in readout FAs, directly influence calculated T_1_ values, requiring accurate B_1_^+^ correction and increasing T_1_ value variability [22, 51]. Fast T_1_ evaluation proposed by Look and Locker [52], as well as T_1_ quantification by snapshot-FLASH [53] and related techniques [54, 55], yield only apparent T_1_ values. These values are affected by the applied FA, thus by the B_1_⁺ inhomogeneity, as magnetization is partially attenuated during image acquisition. As a result, these methods require appropriate correction [53] to yield accurate T_1_ quantification.

The sequence used in the present study employs a single snapshot-FLASH readout per inversion pulse (three shots per slice in total), which reduces the sensitivity to B_1_^+^ inhomogeneities and provides high SNR. Both phantom experiments with simulations (Supplementary Information Fig. S1) and in-vivo data demonstrated that the calculated T_1_ values are not affected by B_1_⁺ variations. As shown in Supplementary Information Fig. S1b, an alteration of the readout FA induces a change in a and b coefficients of Eq. 2, whereas the T_1_ remains unaffected. Moreover, T_1_ values remain stable despite inhomogeneous IE, which strongly correlates with B_1_⁺, both in phantom and in-vivo. Adiabatic inversion pulses may be considered in the future to achieve a more homogeneous IE distribution. It should also be noted that the IE estimated in the fitting model represents an apparent value, as it is biased relative to the actual IE due to saturation effects and may depend on T_1_ and T_2_. A similar observation has been reported for the saturation recovery single-shot acquisition scheme, known as SASHA [56], which uses a single balanced steady state free precession snapshot readout following a 90° saturation recovery pulse. However, that this low sensitivity to B_1_⁺ inhomogeneities does not apply to regions with severe B_1_⁺ dropouts (relative B_1_⁺ < 0.4), highlighting the necessity of prior B_1_⁺ shimming. On the other side, too high B_1_^+^ values yield high readout FAs that lead to signal attenuation due to readout-induced magnetization dampening. As a result, this attenuation can introduce image blurring, as demonstrated in the Shepp–Logan simulation (Supplementary Information Fig. S2). These findings suggest that the actual readout FA should be kept below 20. Additionally, in-flow may bias T₁ values, particularly in vessels. To minimize this effect, a non-selective inversion recovery pulse was used.

Nevertheless, several limitations of our study should be acknowledged. First, the proposed T_1_ mapping approach is relatively time-consuming, requiring approximately 6 min to acquire a single 2D T_1_ map. While this acquisition time may be suboptimal for routine use, the high accuracy and reproducibility of the results make the technique well-suited for reference T_1_ assessment in the abdomen, which was the purpose of this work.

In addition, in this study a TR of 7000 ms was used, resulting in a maximum single breath-hold time of 21 s, which is close to the upper edge of the comfortable range for most subjects. Since TR could not be substantially increased, it remained shorter than 5 × T_1_ for some tissues (e.g., kidney medulla), which could in principle lead to ghosting artifacts. Nevertheless, as shown in Supplementary Information Fig. S3, the impact on the calculated T_1_ values was less than 0.1% even in the worst case (T_1_ = 2700 ms, approximate value for kidney medulla), indicating that the proposed T_1_ mapping approach is effectively insensitive to such ghosting under the present conditions.

Furthermore, although the snapshot readout enables breath-hold acquisitions that reduce motion artifacts, variations in breath-hold position may still influence the T_1_ fitting process. However, masking of the T_1_ maps based on fitting accuracy ensures the reliability of the reported data. Third, in this work only a limited number of subjects was included and a larger cohort studies are therefore required to validate and extend the present findings.

In conclusion, this study provides reliable T_1_ values at 7T for the liver, skeletal paraspinal muscle, kidney cortex, kidney medulla, and, for the first time, the spleen. This was achieved using a B_1_^+^-insensitive T_1_ mapping approach in combination with static pTx, enhancing both the accuracy and repeatability of the measurements. Future studies with larger and more diverse cohorts of healthy volunteers and patients, as well as inclusion of additional abdominal organs such as the pancreas, are needed to further validate and extend these findings. The measured T_1_ values have the potential to serve as reference standards for the development of faster and more advanced T_1_ mapping techniques, as well as for MR simulations and quantitative modeling.

## Supplementary Information

Below is the link to the electronic supplementary material.

Supplementary file1 (DOCX 1705 KB)

## Data Availability

The datasets analysed during the current study are not publicly available due to privacy concerns. The software for data processing is available from the corresponding author upon a reasonable request.
