# Supplementary material for: Abdominal T1 relaxation times at 7T
Source: MAGMA. 2026 Jan 3;39(4):605–17. doi: 10.1007/s10334-025-01317-4 (PMC13447480; doi:10.1007/s10334-025-01317-4)
Supplement: Supplementary file 1 [file 10334_2025_1317_MOESM1_ESM.docx]

**Supplementary information**

**Abdominal T_1_ relaxation times at 7 T**

Petr Bulanov^1,2^, Petr Menshchikov, Johannes A. Grimm, Niklas Himburg, Simon Schmidt, Stephan Orzada, Mark E. Ladd and Sebastian Schmitter

^1^Division of Medical Physics in Radiology, German Cancer Research Center (DKFZ), Heidelberg, Germany.

^2^Faculty of Physics and Astronomy, Heidelberg University, Heidelberg, Germany.

**Journal:** Magnetic Resonance Materials in Physics, Biology and Medicine

**Corresponding author:** Petr Bulanov, [peter.a.bulanov@gmail.com](mailto:peter.a.bulanov@gmail.com), +4917659706329

Simulation of M_z_ evolution during ideal recovery and readout was performed based on following equations:

$$M_{z}^{0}=-1; M_{z}=1-2e^{{-t}/{T_{1}}} , \left[ 1 \right]$$

$$M_{z}^{i}=1+\left[ M_{z}^{i-1}\cos\left( \alpha\right)-1 \right]e^{{-TR\_pulse}/{T_{1}}} , [2]$$

where Equation 1 describes ideal $M_{z}$ recovery after 180˚ inversion ($M_{z}^{0}=-1)$ and Equation 2 represents $M_{z}$ after $i$-th readout pulse with flip angle $\alpha$ and readout interpulse delay $TR\_pulse$. After readout, $M_{z}$ recovery continuous during time $TR-TI-TR_{pulse}*N$, where $TI$ – inversion time, $N (=36)$ is the number of readout pulses in one snapshot and $TR (=7000 ms)$ is sequence repletion time between two inversions (e.g. readouts).

Supporting Information Figure S1a demonstrates simulated M_z_ evolution for T_1_ = 2000 with sequence parameters: $TR=7000 ms$, $TI=3000 ms$, $FA=\alpha=12^{\circ}$. $TR<5*T_{1}$ makes $M_{z}^{0}> -1$ for second and third inversion recovery epoch.

The simulation was performed for three FA = 5°, 10°, 15° and mean M_z_ values, which corresponds to k-space center, were approximated to calculate T_1_, $a$, $b$ using following equation:

$$S_{n}=a+be^{-\frac{{TI}_{n}}{T1}} [3]$$

Results (Supporting Information Figure S1a) demonstrate that calculated T_1_ is independent from FA, which affects only the coefficients $a$ and $b$.

Additionally, the described simulation algorithm was applied for Shepp–Logan phantom. Supporting Information Figure S2 demonstrates Shepp–Logan phantom with T_1_ = 1500 ms signal simulation for three different readout FAs (sequence parameters: TR = 7000 ms, TI = 300, 600, 1000, 2000, and 6000 ms, and FA = 5°, 15°, and 30°). A high FA of 30° results in significant signal reduction due to the readout dampening effect and also leads to blurring effects.

Supporting Information Figure S3 demonstrates Shepp–Logan phantom simulations showing signal evolution and T_1_ maps for three different repetition times (TR = 5000 ms, 7000 ms, and 15000 ms) with a constant phantom T_1_ of 2700 ms (approximate value for kidney medulla). For TR < 5 × T_1_, ghosting artifacts appear, which disappear as TR increases. However, the difference in mean T_1_ across the entire phantom between TR = 7000 ms and TR = 15000 ms is 0.1%, which indicates that proposed T_1_ mapping approach is insensitive to the ghosting artifacts for TR = 7000 ms and maximal measured T_1_ = 2700 ms (the worst case, measured in kidney medulla).

**Supplementary Information** **Fig. S1**. A) M_z_ evolution during GRE-IR sequence for T_1_ = 2000, sequence parameters: TR = 7000 ms, TI = 3000, FA = 12°. It is demonstrated that $TR<5*T_{1}$ makes $M_{z}^{0}> -1$ for second and third inversion recovery epoch. b) Inversion recovery curves, simulated for TI = 300, 600, 1000, 2000, 3000, 4000, 5000 and 6000 ms and FA = 5° (blue line), 10° (red line) and 15° (green line). Calculated T_1_ is independent from FA, which affects only the coefficients $a$ and $b$.

**Supplementary Information Fig. S2.** Shepp–Logan phantom signal simulation of the GRE-IR sequence used in the study, with T_1_ = 1500 ms, TR = 7000 ms, TI = 300, 600, 1000, 2000, and 6000 ms, and FA = 5°, 15°, and 30°. A high FA of 30° results in significant signal reduction due to the readout dampening effect and also leads to blurring effects.

**Supplementary Information Fig. S3.** Shepp–Logan phantom simulations showing signal evolution and T_1_ maps for three different repetition times (TR = 5000 ms, 7000 ms, and 15000 ms) with a constant phantom T_1_ of 2700 ms (approximate value for kidney medulla). For TR < 5 × T_1_, ghosting artifacts appear, which decrease as TR increases. The difference in mean T_1_ across the entire phantom between TR = 7000 ms and TR = 15000 ms is 0.1%. T_1_ maps were calculated using wider range of TI, then shown on the image (TI = 300, 600, 1000, 2000, 3000, 4000, 5000 and 6000 ms)

**Supplementary Information Fig. S4.** GRE-IR images of the liver obtained with different TIs (300 – 6000 ms).

**Supplementary Information Fig. S5.** GRE-IR images of the muscle obtained with different TIs (300 – 6000 ms).

**Supplementary Information Fig. S6.** GRE-IR images of the spleen obtained with different TIs (300 – 6000 ms).
